# Modulation of subjective peripheral sensation, F-waves, and somatosensory evoked potentials in response to unilateral pinch task measured on the contractile and non-contractile sides

**DOI:** 10.1371/journal.pone.0261393

**Published:** 2022-04-22

**Authors:** Terumasa Takahara, Hidetaka Yamaguchi, Kazutoshi Seki, Sho Onodera

**Affiliations:** 1 Department of Sport Social Management, KIBI International University, Takahashi, Okayama, Japan; 2 Department of Human Health and Wellbeing, University of Marketing and Distribution Science, Kobe, Hyogo, Japan; 3 Department of Health and Sports Science, Kawasaki University of Medical Welfare, Kurashiki, Okayama, Japan; BG-Universitatsklinikum Bergmannsheil, Ruhr-Universitat Bochum, GERMANY

## Abstract

Depression of the sensory input during voluntary muscle contractions has been demonstrated using electrophysiological methods in both animals and humans. However, the association between electrophysiological responses of the sensory system and subjective peripheral sensation (SPS) during a voluntary muscle contraction remains unclear. This study aimed to describe the changes in SPS, spinal α-motoneuron excitability (F-wave to M-wave amplitude), and somatosensory evoked potentials (SEPs) during a unilateral pinch-grip task. Outcome variables were measured on the side ipsilateral and contralateral to the muscle contraction and at rest (control). Participants were 8 healthy men aged 20.9±0.8 years. The isometric pinch-grip task was performed at 30% of the maximum voluntary isometric force measured for the right and left hands separately. The appearance rate of the F-wave during the task was significantly higher for the ipsilateral (right) hand than for the contralateral (left) hand and control condition. Although there was no difference in the F-wave latency between hands and the control condition, the amplitude of the F-wave was significantly higher for the ipsilateral (right) hand than for the contralateral (left) hand and the control condition. There was no difference in the amplitude of the SEP at N20. However, the amplitude at P25 was significantly lower for the ipsilateral (right) hand than for the contralateral (left) hand and the control condition. The accuracy rate of detecting tactile stimulation, evaluated for 20 repetitions using a Semmes–Weinstein monofilament at the sensory threshold for each participant, was significantly lower during the pinch-grip task for both the ipsilateral (right) and contralateral (left) hands than in the control condition. Overall, our findings show that SPS and neurophysiological parameters were not modulated in parallel during the task, with changes in the subjective sensation preceding changes in electrophysiological indices during the motor task. Our findings provide basic information on sensory-motor coordination.

## Introduction

When the peripheral nerves are electrically stimulated, the ascending afferent input is projected to the somatosensory cerebral cortex via the spinal cord, and the resulting cortical somatosensory evoked potentials (SEPs) can be recorded. During voluntary muscle contraction, sensory information induced by the electrostimulation of the nerves supplying the contracting muscle is inhibited, and the amplitude of SEPs decreases [1–4]. This suppression of the sensory potential is known as “gating.” The amount of gating during voluntary movement is dependent on the difficulty of the movement [5] and is observed during the pre-movement and movement phases [6, 7]. Based on these findings, the primary functional role of gating is to eliminate unnecessary sensory information during the execution of purposeful voluntary movements.

H-reflexes and F-waves are often used as a tool to investigate spinal excitability and its role in human motor control. H-reflex is derived from the ascending inputs through Ia sensory nerve fibers that excite the monosynaptically connected spinal anterior horn cells, which conduct antegrade muscle action potentials to α-motoneurons. F-wave is derived from the retrograde impulses that are generated by supramaximal stimulation, which excite the spinal anterior horn, and the antegrade conduction that is generated again on the motor nerve fibers and reaches the muscle. Studies using the H-reflex [8] and F-wave [9, 10] have shown an increase in the excitability of spinal α-motoneurons innervating the active muscles during voluntary muscle contraction of the upper and lower limbs. The excitability of spinal α-motoneurons has also been shown to increase with the contractions of distal [11] and contralateral [9] muscles. SEP gating has also been observed in the primary sensory cortex on the side contralateral to the active muscle contraction [12], although there is no consensus on this finding [13]. Although the depression of sensory input during voluntary muscle contraction has been demonstrated using electrophysiological methods in both animal and human studies, the association between the electrophysiological response of the sensory system and subjective peripheral sensation (SPS) during an active muscle contraction remains unclear.

In a previous study, we reported a reduction in the cutaneous sensation on the dorsal surface of the hand during an isometric pinch-grip task under submaximal conditions compared to a no-motion (rest) condition [10]. However, it is not clear whether this response occurred locally only in the hand on the side of the contraction or would also be observed on the non-contracting side. Therefore, this study aimed to evaluate the changes in SPS, spinal α-motoneuron excitability, and SEPs on the side ipsilateral and contralateral to an active contraction of a hand muscle, the right abductor pollicis brevis (APB).

## Materials and methods

### Participants and statement of ethics

The study group included 8 healthy adult men (mean ± SD age, 20.9± 0.8 years; height, 170.0 ±4.5 cm; and weight, 66.3 ± 9.5 kg) with no history of *neurological* diseases. Five participants were right-handed, and the other three were left-handed. The dominant arm was defined as the hand used for writing. Our study was conducted in accordance with the principles of the Declaration of Helsinki. Written Informed consent was obtained from all participants prior to participation. The study was approved by the Ethical Review Board of Kibi International University (No. 20–41).

### Electrical stimulation protocol

A square wave pulse, 0.2 ms in duration, was applied to the median nerve in the area of the carpal tunnel of the right hand using a surface electrostimulation apparatus (NM-420S, Nihon Kohden, Japan) to stimulate the APB. The stimulation electrodes were placed 20 mm apart, with the cathode on the proximal side and the anode on the distal side. The stimulation electrodes were secured using a fixation band to regulate the pressure applied to the electrodes. The stimulation sites that can induce the M-wave of APB with minimal electrical stimulation intensity were identified.

## F-wave recording and analysis

Surface electromyogram (EMG) during electrostimulation was recorded over the right APB (the side of stimulation at the carpal tunnel) using Ag/AgCl bipolar electrodes (5 mm diameter, 20 mm interelectrode distance; Nihon Kohden, Japan). The recording electrode was applied over the muscle belly of the APB, with the reference electrode placed over the first proximal phalanx. The ground electrode was placed in the palm of the right hand. The position of each electrode was fixed with surgical tape. Standard skin preparation was performed prior to electrode placement: the site was cleaned with rubbing alcohol, and the skin was abraded using sandpaper to achieve a skin resistance of <5 kΩ. An EMG/evoked potential testing device (Neuropack MEB-9404, Nihon Kohden, Japan) was used. F-waves were recorded using a band-pass filter (1.5−3 kHz) at a sampling frequency of 10 kHz. To obtain the maximum M-wave, the intensity of electrostimulation was set to 1.2× the intensity at which the M-wave amplitude of the right APB peaked out. The stimulation frequency was set to 1 Hz and was applied for approximately 30 s. The following parameters of the F-wave were calculated during electrostimulation: appearance rate (%), latency (ms), and amplitude of the F/M ratio (%). The appearance rate was the number of F-waves observed on the monitor (threshold, 500 μV/D) from a total of 30 possible waves generated by electrostimulation. Latency was quantified as the average time from electrostimulation to F-wave initiation. The amplitude of the F-wave was expressed as the ratio of the average peak-to-peak amplitude of the F-wave to the maximum M-wave amplitude.

### SEP recording and analysis

Based on the international 10–20 system, the SEPs were recorded from the somatosensory area of the right upper arm (C3’; 2 cm posterior to C3) on the side contralateral to the electrostimulation. The reference electrode was placed at point Fz. The ground electrode was placed on the right forearm. Electrodes were attached to the skin surface using a conductive paste, with a skin resistance of <5 kΩ after standard preparation. The electrostimulation intensity was set to just above the motor threshold, with a stimulation rate of 3 Hz. An EMG/evoked potential testing device (Neuropack MEB-9404, Nihon Kohden, Japan) was used, and SEP waveforms were recorded using a band-pass filter (20 Hz to 10 kHz) at a sampling frequency of 10 kHz, with 200 responses averaged. SEP waveforms were evaluated for 100 ms, both at the time of electrostimulation and 100 ms after stimulation. Epochs with artifacts due to eye movements or blinking (> ±6 μV from baseline) were excluded automatically before averaging. A plate electrode was used to record the evoked electroencephalogram (Ag/AgCl electrode, NE-132B (Φ, 10 mm), Nihon Kohden, Japan). The peak-to-peak amplitude of the SPE at N20 and N20-P25 from baseline, which are the early components after electrostimulation, were analyzed.

### SPS measurement

Prior to the experiment, the SPS threshold on the dorsal surface of the right hand was measured using the Semmes-Weinstein monofilament test (SAKAI Medical Co., Ltd., Tokyo, Japan) [10] without muscle contraction. Filament stimulation was applied over the skin surface against the connection between the first and second metacarpals on the dorsal hand. This area is innervated by C6 according to the dermatome. Monofilament stimulation was performed using gradually thicker filaments, starting from thin filaments of 0.008 g. Confirmation tests were repeated approximately five times for each intensity, and the filament thickness that could be sensed correctly at a rate of approximately 100% was defined as the SPS threshold. The experimenter lowered the filament vertically onto the hand, removed it, and returned it to its original position within 1 s. After establishing their SPS threshold intensity, participants reported the presence or absence of subjective cutaneous sensation to the stimulation at that intensity for each experimental condition. The stimulation interval of the filament was random for more than 1 s and was repeated 20 times. Participants were instructed to give verbal cues when they sensed filament stimulation, and the accuracy rate of detection was calculated. All measurements were performed by the same experimenter. The filament stimulation site was marked to avoid experimenter error resulting in random deviation in measurements due to a large stimulation site.

### Experimental procedure

Participants were seated in a chair with both arms placed on the armrests, with their eyes open for performing a pinch-grip task. When the pinch-grip task was performed with the right hand, the left arm and hand were maintained in a neutral position (no-motion, at-rest condition). When the pinch-grip task was performed using the left hand, the right arm and hand were positioned in the neutral position. Before performing the task, the maximum voluntary isometric force (MVIF) was measured separately for the right and left hands from which the target force level was calculated. To calculate the MVIF, participants held the pinch force meter with the thumb and index finger and were asked to exert their maximum pinch force and to hold this force for 5 s. The peak force measured over this 5 s epoch was defined as the MVIF. After a sufficient rest period (≥10 min), the experimental task was performed. Participants were asked to maintain a 30% MVIF for a duration of 2 min, with the target pinch force to be exerted displayed visually on a computer screen placed 1 m in front of participants. The task was performed with both the right (ipsilateral to the side of recording) and left hands (contralateral to the side of recording). In the control condition, no pinch force was exerted. The sequence of conditions was randomly selected across participants.

### Statistical analysis

All the values are presented as means ± standard deviation. Differences in measured parameters between the conditions were evaluated using a repeated-measures analysis of variance. The sphericity of the data was evaluated using Mauchly’s test, with Greenhouse-Geisser-corrected significance values being used when sphericity was not met. Post-hoc analysis was performed using Tukey’s test for multiple comparisons. The statistical significance level was set at 5% (P<0.05) for all analyses. All analyses were performed using GraphPad Prism (version 8.3.1 for Macintosh).

## Results

**Fig 1:**
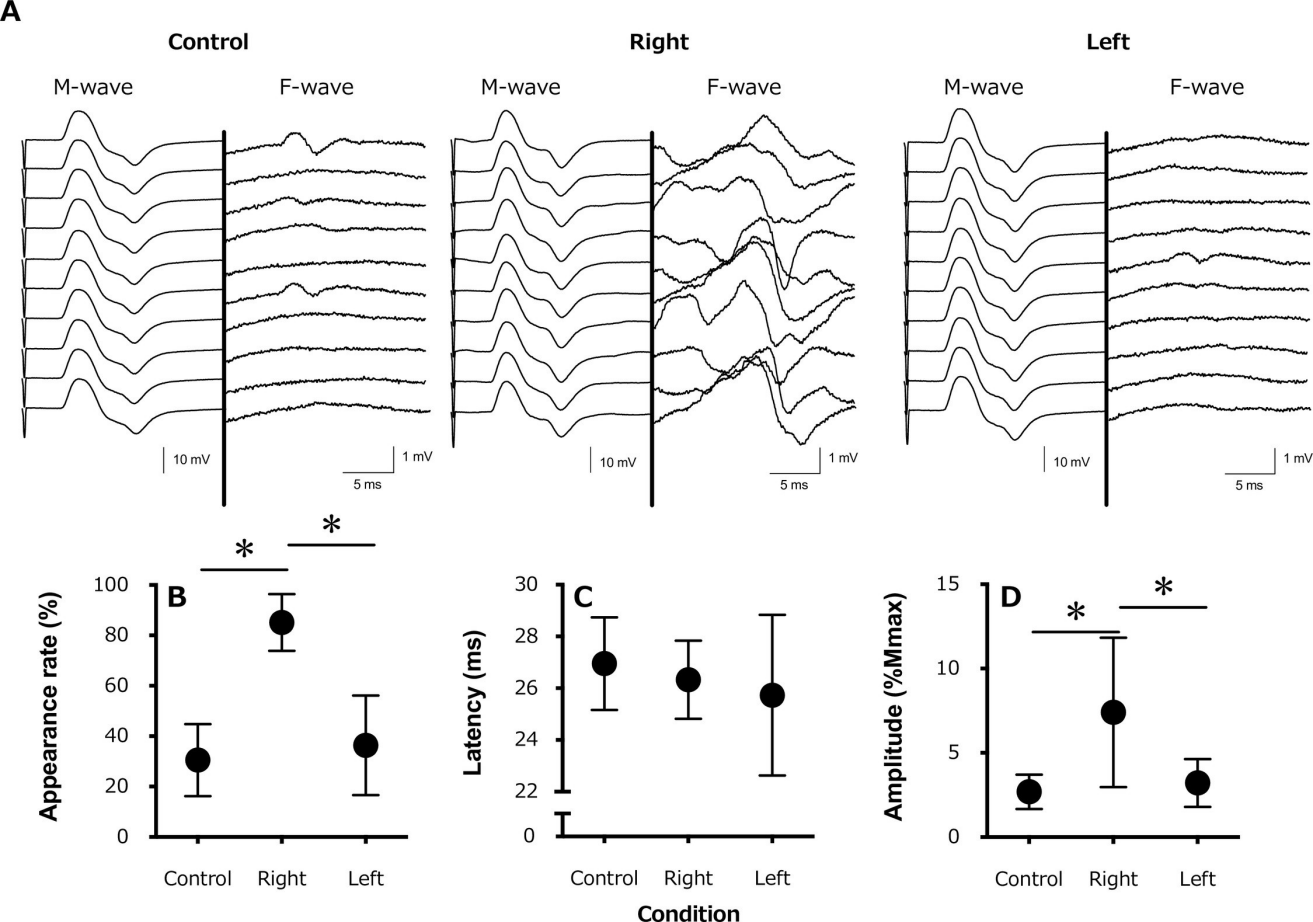
A typical example of an induced electromyogram waveform (10 stimulations) from a single participant in each condition. (A) The waveforms for the M-waves and F-waves are shown on the left and right side of the central thick line, respectively, with the amplitude scaling being 10× higher for the F- than M-wave for the control (rest), active contraction (right) side, and contralateral (left) side; (B) average appearance rate of the F-wave appearance; (C) F-wave latency, and (D) F-wave amplitude. *, P < 0.05.

A typical example of the M- and F-waves is shown in Fig 1A. The appearance rate of the F-wave (Fig 1B) was significantly higher for the side ipsilateral to the SEP recording (right hand, 85.1±11.3%) than for the contralateral side (left hand, 36.4±19.8%) and control (30.5 ±14.3%) condition (F_1.811,12.68_ = 39.78, P<0.01). There were no significant differences in the F-wave latency (Fig 1C) between the control condition (27.0±1.8 ms), the ipsilateral side (right hand, 26.3±1.5 ms), and the contralateral side (left hand, 25.7±3.1 ms; F_1.523,10.66_ = 1.049; P = 0.36). Similar to the appearance rate, the F-wave amplitude (Fig 1D) was significantly higher for the ipsilateral side (right hand, 7.4±4.4%) than for the contralateral side (left hand, 3.2±1.4%) and control condition (2.7±1.0%; F_1.060,7.419_ = 8.206; P = 0.02).

**Fig 2:**
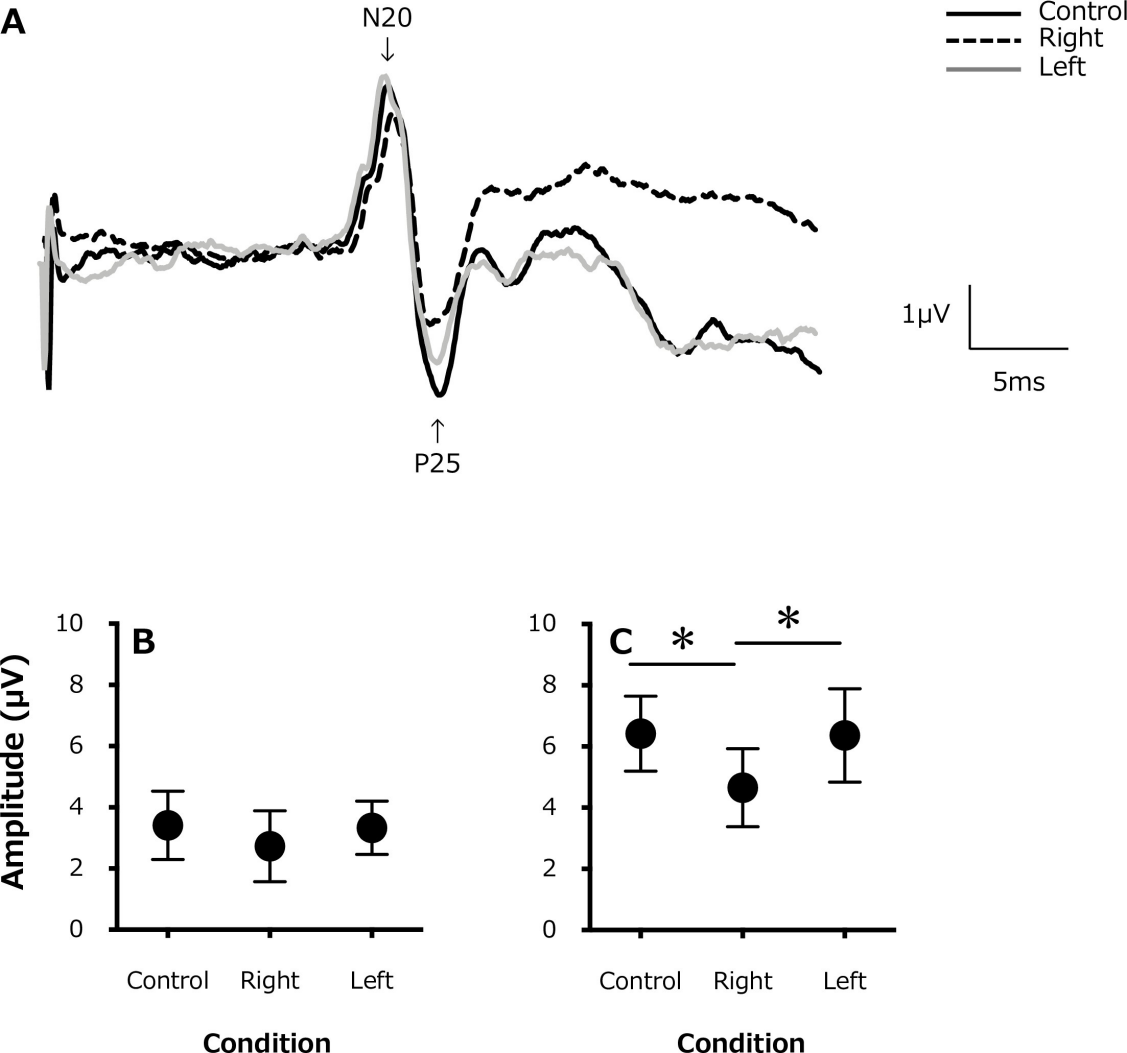
(A). A typical example of somatosensory evoked potentials (SEPs) waveforms from a single participant (for 200 stimulations) for the control (rest) condition (solid black line), in the active contraction (right) side (black dashed line), and contralateral (left) side (gray solid line). Changes in the average SEP amplitude (B) at N20 and (C) P25. *, P < 0.05.

A typical example of SEP waveforms is shown in Fig 2A. There were no differences in the SEP amplitude at N20 (Fig 2B) between the control condition (3.41±1.11 μV), ipsilateral side (right hand, 2.73±1.16 μV), and contralateral side (left hand, 3.33±0.87 μV; F_1.247,8.726_ = 3.222; P = 0.10). However, the SEP amplitude at P25 (Fig 2C) was significantly lower for the ipsilateral side (right hand, 4.65±1.27 μV) than for the contralateral side (left hand, 6.36±1.52 μV) and the control condition (6.42±1.22 μV; F_1.478,10.34_ = 14.63; P = 0.001).

**Fig 3:**
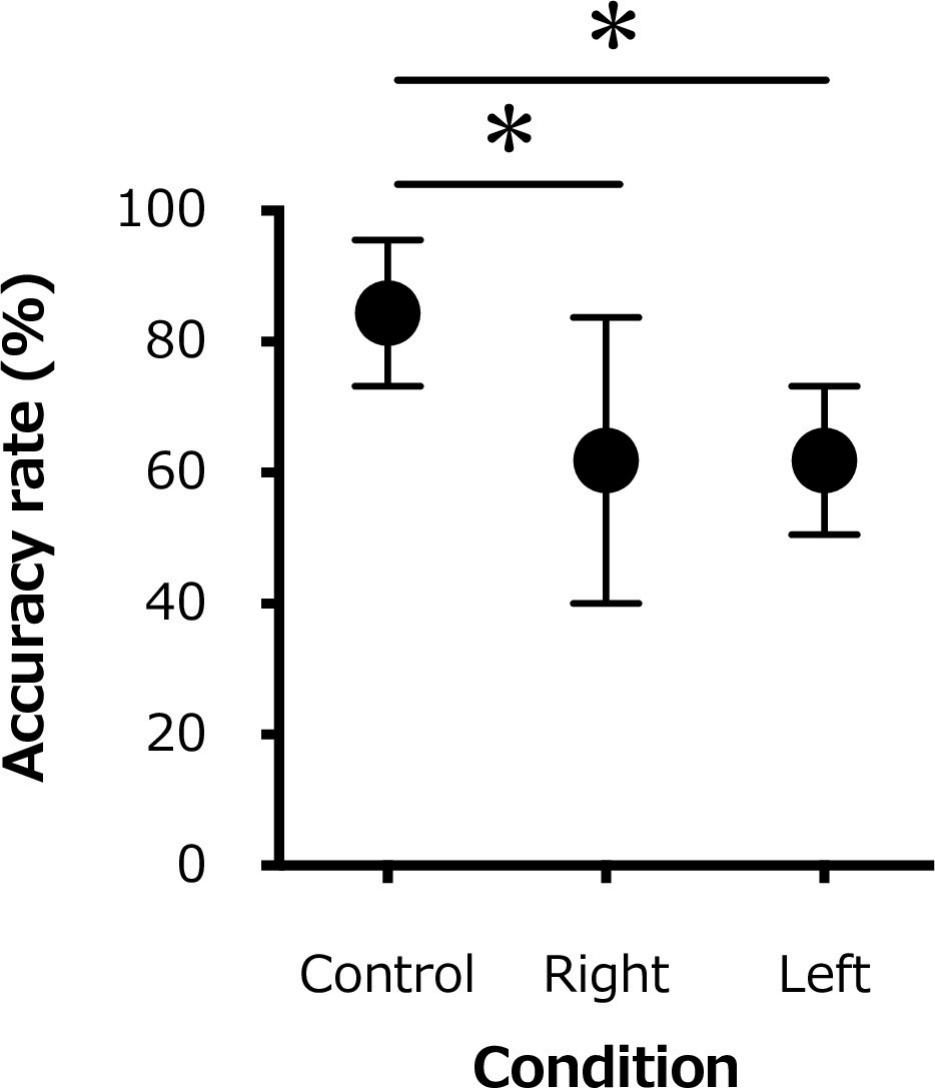
Changes in the average accuracy rate of detection for the 20 repetitions of the monofilament test of all participants for the control (rest) condition, the active contraction (right) side, and the contralateral (left) side. *, P < 0.05.

The accuracy rate for the 20 repetitions of monofilament stimulations was significantly lower for the ipsilateral (right) hand (61.9±21.9%) and the contralateral (left) hand (61.9±11.3%) than for the control condition (84.4±11.2%; F_1.297,9.080_ = 8.158; P = 0.01; Fig 3).

## Discussion

A novel observation of our study was that the pinch-grip task significantly reduced the SPS with active contraction of the right hand on both the ipsilateral (right) and contralateral (left) sides compared with the control condition, although a significant increase in the appearance rate and amplitude of the F- wave and a significant decrease in the amplitude of the SEP (P25) were observed only on the ipsilateral side.

F-waves are muscle action potentials recorded during the electrostimulation of a peripheral nerve that causes retrograde conduction within the axon of an α-motoneuron, followed by subsequent anterograde conduction through the automatic firing of the α-motoneuron in the anterior horn of the spinal cord. In our study, the F-wave appearance rate for the right APB increased during the isometric pinch-grip task performed by the right hand (ipsilateral to the cortical recording side). The F-wave appearance rate indicates the number of motor units participating in the waveform [14] and naturally varies, even at rest. This natural variation suggests that sensory input influences the recruitment of α-motoneurons. This variation in the F-wave appearance rate increased during the isometric pinch-grip task in the ipsilateral (right) hand, which might reflect suppression of activity within the corticospinal tract, which converges on the spinal anterior motor nerve, and inhibition via higher (cortical) control systems. This regulatory effect was not observed for contractions performed using the contralateral (left) hand, with no increase in the appearance rate of the F-wave for the left hand. This finding is different from the previous reports of similar modulation of the F-wave on both the ipsilateral and contralateral sides, with this difference likely reflecting differences in the motor task performed. While we used a pinch-grip task, previous studies used a hand-grip task, with a greater force generated by the hand-grip than the pinch-grip task, increasing the firing rate and recruitment of α-motoneurons [15–17]. We do note that another study reported increased responsiveness of neurons in the primary motor cortex for a precision (pinch-grip) rather than gross (power grip) motor task [18]. The increased muscle recruitment during a power grip task compared with a pinch-grip task complicates the information measured from the upper motor centers due to the integrated processes of the central nervous system. These integrated processes exert an inhibitory effect on the α-motoneurons of the spinal anterior horn for the muscles on the contralateral (non-contraction) side.

The source of SEP at N20 is believed to be the 3b area of the primary somatosensory cortex, representing the stage when the sensory stimulation reaches the primary sensory cortex via the thalamus [19], whereas the source of SEP at P25 is believed to be higher than the 3b area [20]. Therefore, the submaximal isometric pinch-grip task performed with the right hand in our study caused a suppression of the ipsilateral somatosensory input at a higher level than the 3b area. Previous studies on SEP gating during voluntary movement have reported an absence of gating in components corresponding to N20, which is consistent with the findings from previous studies. For these reasons, although the electrophysiological input that is projected to the primary somatosensory area is the same for a given amount of physical stimulation (regardless of the presence or absence of the motor task), this electrophysiological input is suppressed during the subsequent more complex phase of information processing. Additionally, as the amplitude of all components of the SEPs was not different between the left (contralateral) hand and the control (rest) condition, it appears that the electrophysiological sensory input is projected from the periphery to the primary somatosensory cortex without an influence from the contralateral muscle contraction. A previous study reported that the amount of SEP gating was greater in the position task (production of a constant force against a rigid restraint) than in the force task (maintenance of the position against a constant load) despite both tasks generating a similar net muscle torque [5]. This result suggests that more proprioceptive information is required in position tasks than in force tasks and that the sensorimotor regulation may differ depending on the load type due to the complexity of the task during submaximal isometric contraction. The task of maintaining pinch strength at 30% MVIF used in this study may cause cognitive modulation in higher somatosensory regions, which may modulate higher cortical levels.

Various sensory and motor information, such as somatosensory and visual information, must be integrated during motor control. It has been reported that the parietal lobe plays a role in the integration of visual and somatosensory information [21], and more recently, findings on the involvement of the secondary somatosensory cortex have been presented [22]. The secondary somatosensory cortex is known to be strongly influenced by attention [23], although its somatotopic organization is less clear than that of the primary somatosensory cortex. It is possible that the results of changes in activity in these integrative areas are expressed in the body as changes in subjective peripheral sensation, which may contribute to the acquisition of more precise movements.

In our study, we used the accuracy rate for cutaneous stimulation at the sensory threshold in the right hand as an index of SPS. The accuracy rate for cutaneous stimulation was reduced by submaximal isometric muscle contraction in both the ipsilateral (right) and contralateral (left) hands compared to the control (rest) condition. This decrease in the accuracy rate resulted from increased difficulty in recognizing the sensory stimulation. There would be a need to develop a test in which the sensory information can be correctly recognized during a voluntary muscle contraction. Compared to the resting state, transient exercise has been shown to reduce skin temperature sensation [24], with the kinetic threshold also being lower during active muscle contraction than at rest [25]. Therefore, the tactile threshold of the skin surface may increase in the presence of motor output, such as voluntary muscle contraction. Furthermore, the sensory thresholds with a muscle contraction may vary to a certain degree, both increasing or decreasing in amplitude. This phenomenon is also observed during muscle contraction on the contralateral side of the filament stimulation. These results suggest that localized muscle contraction modulates the SPS even in areas that are not related to a muscle contraction or movement.

The primary limitation to the generalization of our findings is that the F-wave, SEP, and SPS measurements were conducted in separate sessions and not measured simultaneously in real-time. As such, identifying the electrophysiological parameters corresponding to the correct and incorrect results on the SPS test was not possible. Real-time measurement of the F-waves, SEP, and SPS in future research would clarify the association between sensory-motor processes and subjective sensory changes in future studies. Another point concerns the age of the participants in this study. The results obtained in this study are from the experiments conducted on young participants. Since aging changes the human sensory perception, there is a possibility that different results could be obtained for SPS changes associated with muscle contraction if different age groups were studied. Nevertheless, this study’s findings provide basic data on the co-regulation of the sensory and motor systems during motor output, thus contributing to the field of rehabilitation medicine as well as information regarding conditioning and injury prevention during exercise for athletes.

## Conclusion

Overall, our findings show that SPS and neurophysiological parameters were not modulated in parallel during the task, with changes in the subjective sensation preceding changes in the physiological indices during the motor task. Our findings provide basic information on sensory-motor coordination.

## Supporting information

S1 Dataset Raw data. (XLSX)
