# Correction: Modulation of subjective peripheral sensation, F-waves, and somatosensory evoked potentials in response to unilateral pinch task measured on the contractile and non-contractile sides

**DOI:** 10.1371/journal.pone.0314498

**Published:** 2024-11-20

**Authors:** Terumasa Takahara, Hidetaka Yamaguchi, Kazutoshi Seki, Sho Onodera

S1 Dataset is uploaded incorrectly. Please view the correct S1 Dataset below.

## Supporting information

S1 Dataset (XLSX)
